# A Case Report: Acute Rheumatic Fever or Something More?

**DOI:** 10.7759/cureus.36967

**Published:** 2023-03-31

**Authors:** Nevein F Sejeeni, Sumaiah S Alfahmi, Razan M Alzhrani, Maha K Almatrafi, Anwar A Hussain

**Affiliations:** 1 General Pediatrics, Maternity and Children Hospital, Makkah, SAU; 2 Pediatric Medicine, Maternity and Children Hospital, Makkah, SAU; 3 Medicine and Surgery, Umm Al-Qura University, Makkah, SAU

**Keywords:** acute rheumatic fever, rheumatic heart disease, carditis, subcutaneous nodules, jones criteria

## Abstract

Acute rheumatic fever (ARF) is an autoimmune response that may occur after a group A Streptococcus (GAS) infection. Subcutaneous nodules are considered a rare manifestation of acute rheumatic fever with an incidence of 0%-10%. We present a case study of a 13-year-old girl who presented to us with subcutaneous nodules and articular involvement described as a non-migratory polyarticular joint pain involving the small joints of the hands, wrist, elbows, knees, and ankles for three months with poor response to the non-steroidal anti-inflammatory drug (NSAID) Ibuprofen. Accompanied with the presence of carditis, the patient fulfilled three major and two minor criteria of the revised Jones criteria 2015. Therefore, a diagnosis of acute rheumatic fever was made. The child was asymptomatic on subsequent visits, and although the subcutaneous nodules subsided, she will continue to receive penicillin every month for five years. We describe the successful diagnosis and management of a patient with ARF.

## Introduction

Acute rheumatic fever (ARF) is the consequence of an immunological reaction to pharyngitis that is caused by an infection with group A β-hemolytic Streptococcus (GAS) [1]. The incidence of ARF is eight to 51 per 100,000 people worldwide. Children between the ages of five and 15 are most frequently affected [2]. Studies have shown an association of ARF with low socioeconomic status, overpopulation, and rural areas with limited access to healthcare [3]. Carditis, arthritis, Sydenham's chorea, erythema marginatum, and subcutaneous nodules are the five major clinical symptoms of rheumatic fever that were first proposed by Dr. T Ducket Jones in 1944 [4]. Subcutaneous nodules have been shown to have a low occurrence rate, as it is reported to be in between 1% to 21% of cases [5]. Lowering poverty and overcrowding in low-income areas is one of the primary preventative actions and the cornerstone of management. Additionally, studies have demonstrated that administering antibiotics to patients with symptoms suggestive of streptococcal infection can lower the risk of rheumatic fever by as much as 70%. Long-term benzathine penicillin treatment is a secondary prevention measure to avoid recurring rheumatic fever [2].

## Case presentation

A 13-year-old girl presented with complaints of dyspnea, subcutaneous nodules, and joint pain for the last three months. Joint pain developed over three months, affecting her knees, ankles, elbows, wrists, and fingers. The pain was bilateral, non-migratory, on the right side more than the left, and gradually worsened enough to affect her daily activities. The pain was severe during night and dawn, which incapacitated her and led her to skip school. She initially showed improvement with the non-steroidal anti-inflammatory drug (NSAID) Ibuprofen, but eventually, she stopped responding to it. The pain was associated with swelling and erythema around the joints.

After two months of joint pain, the mother noticed small, round, non-tender, skin-colored subcutaneous nodules on both elbows and arms while massaging her daughter to reduce her pain. Over time she started complaining of chest pain and exertional dyspnea that increased with lying down.

The patient had a history of upper respiratory tract infection and high-grade fever (39-40ºC) about two weeks preceding the joint pain, which lasted for a week and improved with antipyretic medications. She visited several hospitals and primary health care before being referred to our hospital to be investigated for rheumatic heart disease. Over the last three months, she has been experiencing joint pain, subcutaneous nodules, and exertional dyspnea. She was admitted to our hospital for management and investigation. However, there was no recent history of palpitations, rash, hemoptysis, lability, or abnormal movement.

On physical examination, she appeared in pain, with a temperature of 37ºC. Respiratory rate, heart rate, and blood pressure were 28/min, 129/min, and 126/70 mmHg, respectively. Her height was 153 cm, weight was 81 kg, and her BMI was 35. Cardiovascular examination was normal except for a grade 3 holosystolic murmur with the highest intensity at the apex.

**Figure 1:**
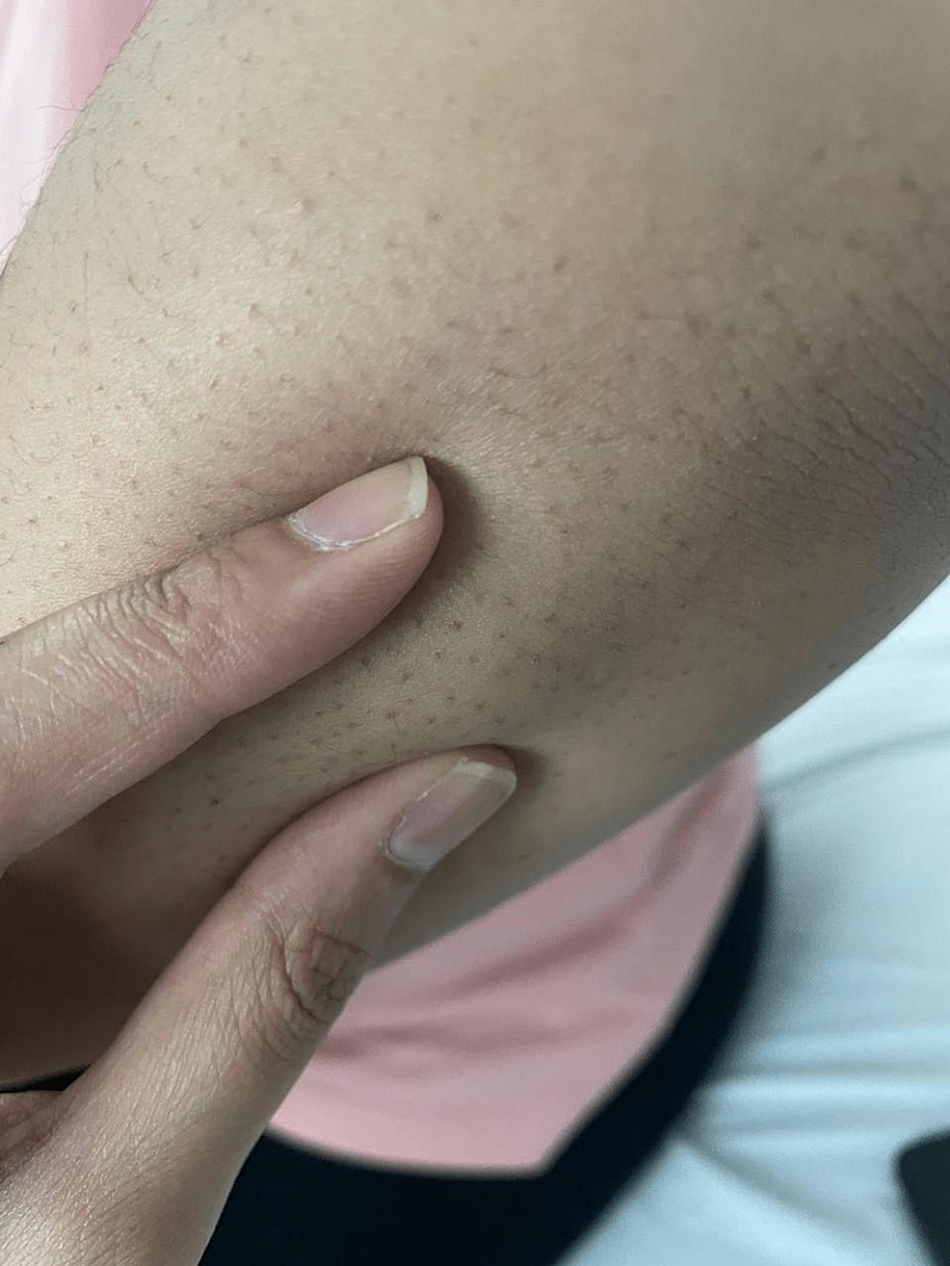
Multiple subcutaneous nodules over the extensor surface of the right elbow.

**Figure 2:**
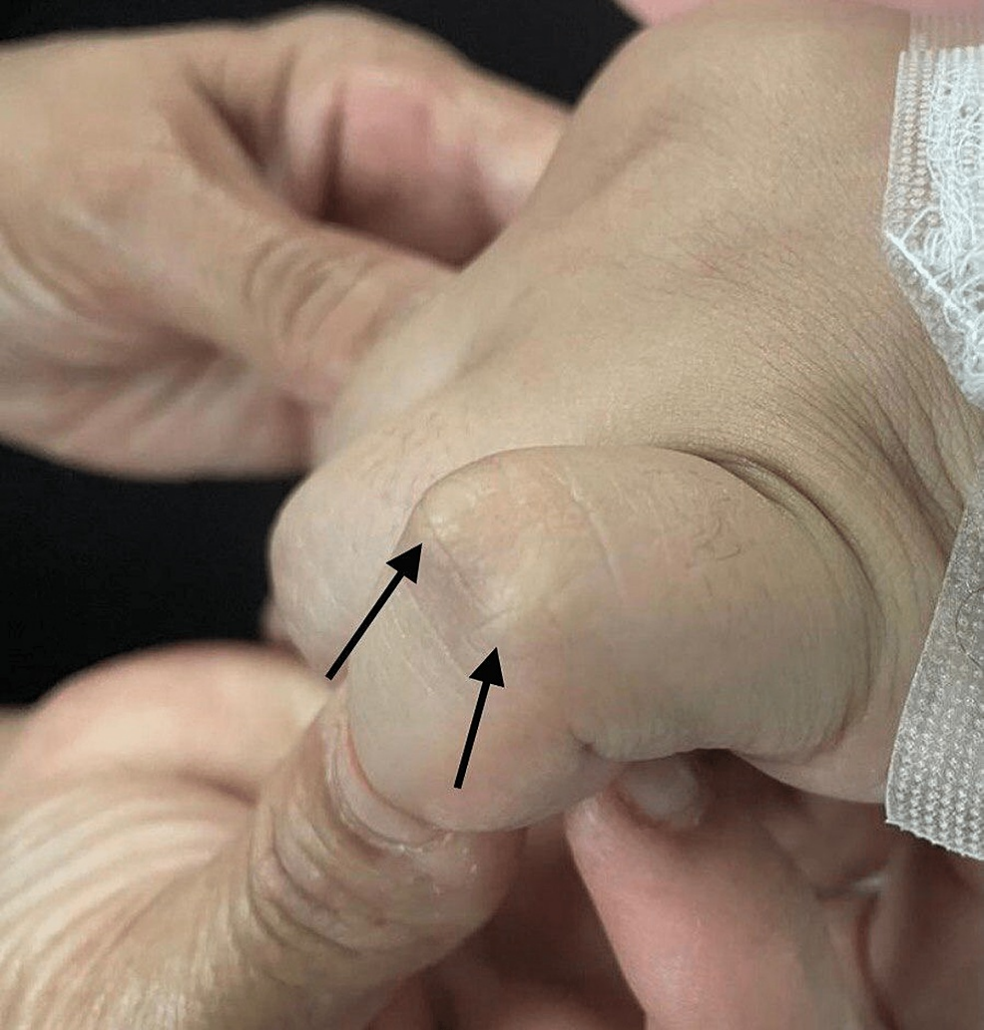
Subcutaneous nodules over the interphalangeal joints.

She had an antalgic gait, subcutaneous nodules at the extensor surface of the right elbow, and interphalangeal joints with no sign of arthritis (Figure 1, 2). Upon investigation, a complete blood count revealed hemoglobin = 11.0 g/dL, white cell count = 13,4*1000 uL, platelets = 483*1000 uL, a blood culture showed no growth, COVID-19 was negative, rheumatoid factors were negative, erythrocyte sedimentation rate (ESR) = 120 mm/hr, C reactive protein (CRP) was 2.3 mg/dl, and throat culture was negative for group A Streptococcus, but she had elevated titers of anti-streptolysin O titer (ASO) (832 IU/ml). Echocardiography revealed severe mitral regurgitation (MR), mild aortic regurgitation (AR), and diffuse mild pericardial effusion. Chest x ray and electrocardiogram (ECG) were normal. Based on the revised Jones criteria for moderate to high-risk populations, she had evidence of a recent streptococcal infection with three major criteria (presence of polyarthralgia, carditis, and subcutaneous nodules) and two minor criteria (fever of ≥38.5°C and ESR of ≥60 mm), for which a diagnosis of acute rheumatic fever was made.

She was treated with intramuscular (IM) 1200000 U of benzathine penicillin every three weeks and will continue for five years with a probability of lifelong use. Captopril 12.5 mg every eight hours, Lasix 40 mg twice a day, and naproxen at a dose of 500 twice a day for three weeks were started. After one month, the nodules disappeared.

## Discussion

Rheumatic fever (RF) is one of the major causes of acquired heart disease in the pediatric population. A significant burden of this disease worldwide affects mainly children between the ages of five and 14. ARF pathophysiology is thought to be an autoimmune cross-reactivity due to molecular mimicry following a group A streptococcal (GAS) infection [6]. The diagnosis is made clinically through the revised Jones criteria (2015), which is summarized in Table 1.

**Table 1:** Revised Jones criteria. The diagnosis of first RF episode required a confirmation of two major criteria or one major and two minor criteria, along with evidence of antecedent group A beta hemolytic streptococcal infection RF, rheumatic fever; CRP, C-reactive protein; ESR, erythrocyte sedimentation rate

Revised Jones criteria (Gewits et al., 2015 [7])	
Major criteria	
Low-risk population	Moderate to high-risk population
Carditis (clinical and/or subclinical), polyarthritis, chorea, erythema marginatum, subcutaneous nodules	Carditis (clinical and/or subclinical), mono- or polyarthritis, polyarthralgia, chorea, erythema marginatum, subcutaneous nodules
Minor criteria	
Low-risk population	Moderate to high-risk population
Polyarthralgia, fever (≥38.5°C), peak ESR ≥60 mm in the first hour and/or CRP ≥3.0 mg/dL, prolonged PR interval	Monoarthralgia, fever (≥38°C), Peak ESR ≥30 mm in the first hour and/or CRP ≥3.0 mg/dL, prolonged PR interval

They are categorized into major and minor criteria, and recently, risk stratification has been used to divide populations into low-risk and moderate to high-risk groups [7]. Moderate to high-risk populations are defined based on ARF incidence of two or more per 100,000 school-aged children or all-age rheumatic heart disease (RHD) prevalence of more than one per 1,000 people per year. Alqanatish et al. reported that the Saudi population is considered one of the moderate to high-risk groups [6]. Our patient met the revised Jones criteria for moderate to high-risk populations (2015), as evidenced by a positive anti-streptolysin O (ASO) titer, carditis as evidenced by echocardiogram results, polyarthralgia, and subcutaneous nodules. She also met several minor criteria, including a high-grade fever and an elevated ESR.

Subcutaneous nodules are a rare manifestation of ARF; their incidence is reported to be 0%-10% [7]. A previous study published in 2009 in Saudi Arabia to recognize the pattern of ARF in children revealed that the most common major manifestations of ARF were arthritis (77% of the cases) and carditis (54% of the cases). Sydenham chorea was present in 20% of the cases. None of the cases presented with subcutaneous nodules or erythema marginatum [6]. A review of the recent literature reveals that patients who presented with subcutaneous nodules had an association with severe carditis [8]. Poonia et al. reported that subcutaneous nodules usually accompany severe carditis and manifest four to six weeks after the beginning of an acute episode [9]. Here we reported a case of acute rheumatic fever in a 13-year-old Saudi female who complained of joint pain associated with subcutaneous nodules and multiple attacks of dyspnoea and chest pain for the last three months. On her examination, she had an antalgic gait and subcutaneous nodules on the extensor surface of the right elbow and interphalangeal joints. The patient underwent echocardiography to investigate for carditis; her results are revealed in the case presentation, which confirmed the diagnosis of ARF. On review of the literature, using adequate antibiotics in confirmed cases is critical for the primary prevention of ARF in GAS infections [1]. A review article on rheumatic heart disease revealed that penicillin is the only effective therapy for Strep A eradication in the pharynx. Additionally, it is recommended as a secondary preventive measure in cases where ARF and rheumatic heart disease have been diagnosed [10]. The main goal of treating ARF after eradication of Strep A is to treat clinical manifestations such as arthritis, carditis, and chorea [6]. Therefore, we used a single dose of IM benzathine penicillin to eradicate GAS carriage and we will continue with same antibiotic every three weeks for secondary prophylaxis. To treat polyarthralgia, we preferred using naproxen for its proven benefit and fewer adverse effects compared to aspirin with similar efficacy [11,12]. We chose not to use corticosteroids due to the lack of evidence of their benefit in improving patients with fulminating rheumatic carditis [13]. Therefore, we preferred symptomatic management with Captopril and Lasix. A subcutaneous nodule does not require treatment and will fade when the carditis is adequately treated [14].

## Conclusions

This is a case of rheumatic fever with cardiac manifestations and subcutaneous nodules, a rare presentation in the pediatric age. The management will depend on her cardiac abnormality, which may require lifelong benzathine penicillin. Her cardiac medication depends on the aspect of her echocardiographic findings. We report this case for the rare occurrence of subcutaneous nodules and recommend always having a high suspicion of acute rheumatic fever in a child presenting with joint pain so as not to delay the diagnosis and, subsequently, the therapeutic interventions that can prevent the progression of the disease.
